# Maladie de Kaposi à localisation broncho-pulmonaire révélant une infection VIH

**DOI:** 10.11604/pamj.2015.22.279.7476

**Published:** 2015-11-23

**Authors:** Amal Sebbar, Nahid Zaghba, Hanane Benjelloun, Abdelaziz Bakhatar, Najiba Yassine

**Affiliations:** 1 Service des Maladies Respiratoires, CHU Ibn Rochd, Casablanca, Maroc

**Keywords:** Maladie de Kaposi, broncho-pulmonaire, infection VIH, Kaposi sarcoma, bronchopulmonary, HIV infection

## Abstract

La maladie de Kaposi (MK) associée au VIH, forme dite épidémique, a été décrite la 1ère fois en 1981 par Hymmes. C'est l'affection maligne la plus fréquente au cours du SIDA. La MK est à l'origine de 10% des atteintes pleuropulmonaires au cours de l'infection par le VIH et 40% des pneumopathies en cas de MK cutanéomuqueuse. Les localisations pulmonaires occupent la deuxième place des atteintes viscérales après la forme digestive. Le diagnostic repose sur des arguments épidémiologiques, cliniques, radiologiques, biologiques, endoscopiques et histologiques. Nous rapportons un cas de MK broncho-pulmonaire compliquant une infection VIH chez un patient présentant une maladie de Kaposi cutanée de découverte fortuite au cours de l'atteinte pulmonaire. Le diagnostic a été retenu après avoir éliminé les maladies opportunistes à tropisme pulmonaire. Le Kaposi pulmonaire constitue l'atteinte la plus grave de la MK-sida et la survie après le diagnostic est courte malgré les thérapeutiques agressives.

## Introduction

La maladie de Kaposi, décrite la première fois par le dermatologue viennois Moritz Kaposi en 1872, est l'affection maligne la plus fréquente au cours du sida. Les localisations thoraciques sont précédées dans la plupart des cas par une atteinte cutanéo-muqueuse ou viscérale en particulier gastro-intestinale [1, 2]. Nous rapportons un cas de maladie de kaposi à localisation broncho-pulmonaire révélant une infection VIH. Le diagnostic de la localisation broncho-pulmonaire est porté sur des arguments cliniques, radiologiques et endoscopiques.

## Patient et observation

**Figure 1:**
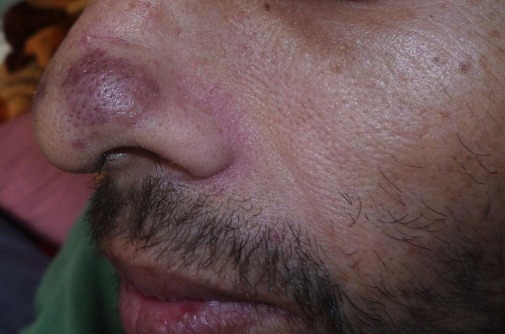
Papules rouges violacées au niveau de l'aile du nez

**Figure 2:**
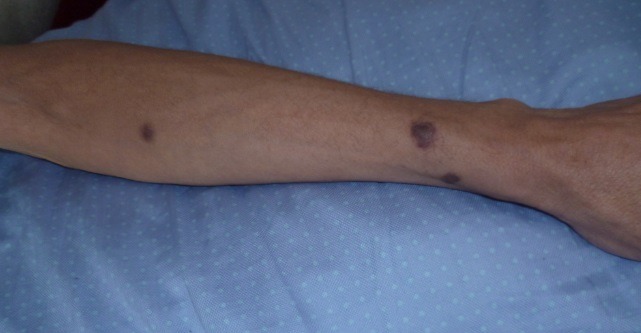
Papules rouges violacées au niveau de l'avant-bras gauche très évocatrices du sarcome du Kaposi cutané

**Figure 3:**
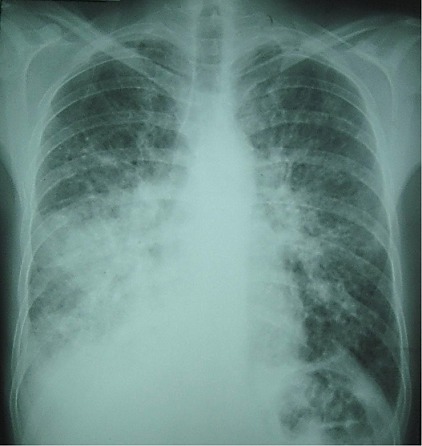
Radiographie thoracique montrant des opacités réticulo-nodulaires bilatérales, confluentes par endroit et prédominantes à la base droite et à la région hilaire gauche

**Figure 4:**
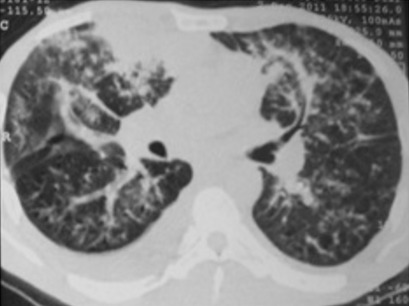
TDM thoracique montrant des foyers de condensation alvéolaire du lobe supérieur droit, des opacités micronodulaires et épaississement des septa péri bronchovasculaires bilatérales, aspect en verre dépoli bilatéral, épanchement pleural droit

**Figure 5:**
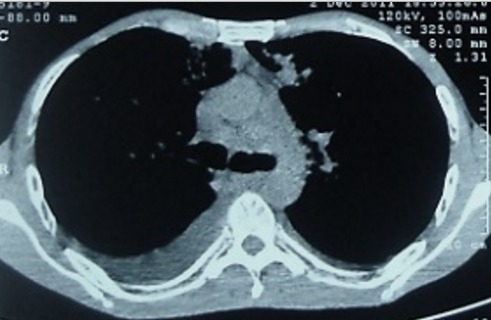
TDM thoracique montrant des adénopathies médiastinales associées à un épanchement pleural droit

Il s'agit d'un patient âgé de 42 ans, tabagique chronique, ayant un comportement sexuel à risque, qui consultait pour une toux sèche d’évolution progressive depuis 5 mois et d'une hémoptysie de faible abondance associée à une dyspnée d'effort d'aggravation progressive et à une altération de l’état général. A l'admission, le patient était polypnéique, en mauvais état général (PS à 2). L'examen pleuro-pulmonaire trouvait des râles crépitants en basal gauche et une matité basale droite. L'examen ORL trouvait une tuméfaction rouge violacée du palais osseux, non douloureuse, évoluant depuis 2 mois. L'examen cutanéomuqueux trouvait des papules et nodules disséminés au niveau du visage, du dos, de la face antérieure du thorax, des deux membres inférieurs et au niveau de la verge, de 0,3 à 3cm, rouges violacés, dures, non douloureux, ne s'effaçant pas à la vitro pression, remontant à 8 mois et augmentant progressivement de taille. Ces lésions faisant évoquer une localisation cutanée de la maladie de kaposi (Figure 1, Figure 2). La radiographie thoracique objectivait des opacités réticulo-nodulaires confluantes par endroit et diffuses aux deux champs pulmonaires avec prédominance aux bases (Figure 3). Le scanner thoracique montrait un foyer de condensation alvéolaire du lobe supérieur droit, des opacités micronodulaires bilatérales et un épaississement des septa péri-bronchovasculaires, un aspect en verre dépoli bilatéral et un épanchement pleural droit (Figure 4, Figure 5). L'hémogramme montrait une anémie hypochrome microcytaire à 9,7g/dl, une lymphopénie à 510/µl. Le test rapide VIH était positif, confirmé par la sérologie VIH avec un taux de CD4 à 27/mm^3^. Les sérologies à cytomégalovirus, toxoplasmose, aspergillaire, hépatite B, hépatite C et syphilitique étaient négatives. Le bilan de la tuberculose était négatif. Le taux de LDH était normal à 226UI/l. La bronchoscopie objectivait une formation tumorale rougeâtre bourgeonnante dès l'entrée de la fosse nasale droite, une lésion rougeâtre à surface plane sur le versant antérieur de la carène et des plages discontinues de lésions rougeâtres à surface plane, étendues sur tout l'arbre bronchique dès l'entrée des 2 bronches principales. Ces lésions évoquaient une localisation bronchique de la maladie de kaposi. La biopsie cutanée avait confirmé la localisation cutanée de la maladie de Kaposi tandis que les biopsies bronchiques étaient non contributives. La recherche de bacilles de Kokh (BK) à l'examen direct et par culture ainsi que la recherche du PneumocystisJiroveci et la culture sur milieu de sabouraud dans le liquide d'aspiration bronchique étaient négatives. Le traitement était basé sur les antirétroviraux (Stavudine (D4T), Lamuvidine (3TC), Efavirenz (EFV)) et une chimiothérapie (Doxorubicine 50 mg/j en cure tous les 19 jours puis Tamoxifène). Une prophylaxie du PneumocystisJiroveci et Toxoplasmose était instaurée à base de TrimitoprimeSulfamétaxazol. L’évolution, après deux mois de traitement, était marquée par une amélioration clinique initiale puis aggravation progressive et décès dans un tableau d'insuffisance respiratoire aiguë.

## Discussion

La maladie de kaposi est l'une des premières maladies opportunistes reconnues du sida. Il s'agit d'une affection tumorale à point de départ multifocale, marquée par une double prolifération endothéliale et fibroblastique anarchique [2]. L'agent causal est l'herpès virus 8 dénommé aussi herpès virus associé au sarcome de Kaposi (KSHV) [3]. La maladie de Kaposi (MK) associée au sida survient préférentiellement chez l'adulte jeune (en moyenne de 35 ans), de sexe masculin (sex-ratio de 20) et dans 70% des cas chez les homosexuels [4]. Les localisations pleuropulmonaires de la MK sont présentes chez près de 45% des patients atteints de MK cutanée, et la présence d'une atteinte pulmonaire est associée dans 85 à 95% des cas à une localisation cutanéomuqueuse [5, 6]. Elles surviennent en général chez des patients sévèrement immunodéprimés [7], comme c’était le cas de notre patient.

L'atteinte pleuro-pulmonaire est souvent asymptomatique et révélée par une radiographie thoracique systématique ou une endoscopie bronchique réalisée dans le cadre du bilan d'une pneumopathie infectieuse associée [2, 5]. Les symptômes sont peu spécifiques: toux, dyspnée inexpliquée, douleur thoracique et hémoptysie [5, 6]. L'absence de fièvre, un début insidieux, des œdèmes déclives mal expliqués ou une localisation cutanée de la maladie sont très évocateurs d'une localisation respiratoire de la MK [2, 5]. Notre patient présentait une toux, des hémoptysies, une dyspnée ainsi que des lésions de kaposi cutané. Cette symptomatologie, peu spécifique, fait évoquer en premier une atteinte pulmonaire de la maladie de Kaposi mais ne permettant pas, toutefois, d’éliminer une origine infectieuse telle une tuberculose. La radiographie thoracique peut être normale comme elle peut mettre en évidence des opacités nodulaires ou des masses uniques ou multiples, denses et homogènes, souvent mal délimitées, pouvant confluer et des opacités linéaires, péri hilaires, bilatérales, prédominant aux bases, se prolongeant en périphérie par des images réticulées plus fines. L'association de ces deux types d'anomalies est très évocatrice [6]. Un épanchement pleural de faible à moyenne abondance, uni ou bilatéral est fréquent [5]. La radiographie thoracique de notre patient montrait des opacités nodulaires bilatérales, confluentes réalisant une opacité basale droite et une autre à projection hilaire gauche avec des opacités linéaires péri-hilaires bilatérales. Le scanner thoracique peut montrer deux formes qui peuvent coexister. Une forme nodulaire constituée de multiples nodules irréguliers, spiculés, à prédominance péri-hilaire et de distribution péri-bronchovasculaire, avec bronchogramme aérique fréquent. Il s'y associe fréquemment des opacités en verre dépoli entourant les nodules et formant un halo. L'autre forme, infiltrante est constituée d’épaississements péri-bronchovasculaires et d’épaississement septaux parfois nodulaires. Des épanchements pleuraux peuvent compliquer les deux formes. Des adénopathies médiastinales ou hilaires peuvent exister chez 30 à 50% des patients [5, 7]. Chez notre patient, la TDM thoracique montrait les lésions des deux formes nodulaire et infiltrante ainsi qu'un épanchement pleural liquidien de faible abondance à droite et des adénopathies médiastinales. Cet aspect scannographique concorde parfaitement avec les données de la littérature. Un taux de CD4 abaissé (< 200 /mm^3^) est généralement observé au cours de l'atteinte pulmonaire [5, 6]. Le taux de CD4 de notre patient était très bas; à 27/ mm^3^.

La confirmation diagnostique est le plus souvent endoscopique avec mise en évidence de lésions sous forme de plaques ou de nodules rouges violines non friables entourées d'un halo hémorragique caractéristique. Ces lésions sont de répartition irrégulière et siègent surtout au niveau de la trachée, des troncs bronchiques et des orifices segmentaires [1, 7]. La certitude diagnostique résulte théoriquement des biopsies bronchiques ou pulmonaires [2]. La biopsie per-endoscopique est souvent négative du fait de la difficulté de pratiquer des prélèvements suffisamment profonds, du siège sous-muqueux profond des lésions et du risque d'hémorragies secondaires sévères [1]. Les biopsies pulmonaires (transbronchiques, transpariétales, mini-thoracotomie) sont invasives [2, 7]. L'analyse du liquide du lavage broncho-alvéolaire (LBA) peut objectiver une hémorragie alvéolaire et permet surtout d’éliminer une infection associée [5, 6]. Ainsi, lorsque le contexte clinique est évocateur (Kaposi cutané prouvé) et après avoir éliminé une infection opportuniste, la visualisation par endoscopie bronchique de lésions rouges ou violacées, généralement très suggestives, est suffisante pour poser le diagnostic [2, 5]. Dans notre cas, la biopsie cutanée a confirmé le Kaposi cutané et la bronchoscopie a permis la visualisation de lésions rouges violines disséminées tout au long de l'arbre bronchique. Le diagnostic de la localisation broncho-pulmonaire de la MK était retenu après avoir éliminé une infection opportuniste par l’étude du liquide du LBA malgré que les biopsies bronchiques aient revenu négatives.

Le traitement de la maladie de Kaposi pulmonaire relève d'association de chimiothérapies cytotoxiques, en complément du traitement antirétroviral [7, 8]. La chimiothérapie, administrée par voie systémique sous forme de mono-ou polychimiothérapie, n'est que palliative permettant une régression rapide et une diminution des symptômes [4, 8]. Les polychimiothérapies associant adriamytine, bléomycine et vincristine sont responsables d'une augmentation du taux de réponse immédiate, mais aussi de la fréquence des infections opportunistes [4]. La monochimiothérapie a été largement utilisée au cours de la dernière décennie avec un taux de réponse globale de 10 à 75% [8]. Parmi les nouveaux traitements, on peut retenir les anthracyclinesliposomales (doxorubicine et daunorubicine), les taxanes et les thérapies ciblées tels les inhibiteurs de la thyrosine kinase dont la talidomide [9]. L'utilisation de la chimiothérapie provoque une immunodépression ce qui justifie la prescription d'une prophylaxie contre la pneumocystose et la toxoplasmose par le cotrimoxazole [5]. En association avec un traitement antirétroviral, notre patient était mis sous monochimiothérapie par doxorubicine avec une prophylaxie contre la pneumocystose et la toxoplasmose. La réponse clinique est habituellement observée en moins de 12 semaines, mais les rémissions complètes n'excèdent que rarement 1 an [8]. Le diagnostic précoce de la MK-SIDA est corrélé à un meilleur pronostic. Ainsi, l'administration précoce d'un traitement antirétroviral permet d'améliorer les résultats du traitement du Kaposi pulmonaire [10]. Le décès survient après 6 à 12 semaines par insuffisance respiratoire aiguë, résultant des lésions spécifiques, des complications de la maladie elle-même et/ou des infections associées [5, 6]. Dans notre cas, le malade est décédé, deux mois après le début du traitement, dans un tableau d'insuffisance respiratoire aiguë.

## Conclusion

La localisation broncho-pulmonaire de la maladie de Kaposi n'est pas exceptionnelle et reste l'infection maligne la plus fréquente au cours de l'infection par le VIH. Le diagnostic est posé devant des arguments biologiques, cliniques, radiologiques et endoscopiques, surtout en l'absence de confirmation histologique souvent difficile à obtenir. Le Kaposi pulmonaire constitue l'atteinte la plus grave de la MK-sida. Son pronostic reste très péjoratif malgré les thérapeutiques mises en route, d'où l'intérêt d'un diagnostic précoce.
